# Role of the rhizosphere bacterial community in assisting phytoremediation in a lead-zinc area

**DOI:** 10.3389/fpls.2022.1106985

**Published:** 2023-01-17

**Authors:** Yunhua Xiao, Liang Chen, Chunxiao Li, Jingjing Ma, Rui Chen, Bo Yang, Gang Liu, Shuming Liu, Jun Fang

**Affiliations:** ^1^ College of Bioscience and Biotechnology, Hunan Agricultural University, Changsha, China; ^2^ College of Chemical and Environmental Sciences, YiLi Normal University, YiLi, China

**Keywords:** Muti-metals contamination, Bioconcentration and translocation factors, Rhizosphere ecological characteristics, Phytoremediation, Bacterial community

## Abstract

Heavy metals (HMs) contamination and vegetation destruction in the mining area caused by mining activities are severely increasing. It is urgent to restore vegetation and stabilize HMs. In this study, we compared the ability of HMs phytoextraction/phytostabilization of three dominant plants, including *Artemisia argyi* (LA)*, Miscanthus floridulus* (LM), and *Boehmeria nivea* (LZ) in a lead-zinc mining area in Huayuan County (China). We also explored the role of the rhizosphere bacterial community in assisting phytoremediation using 16S rRNA sequencing technology. Bioconcentration factor (BCF) and translocation factor (TF) analysis showed that LA preferred accumulating Cd, LZ preferred accumulating Cr and Sb, and LM preferred accumulating Cr and Ni. Significant (*p* < 0.05) differences were found among the rhizosphere soil microbial communities of these three plants. The key genera of LA were *Truepera* and *Anderseniella*, that of LM were *Paracoccus* and *Erythrobacter*, and of LZ was *Novosphingobium*. Correlation analysis showed some rhizosphere bacterial taxa (e.g., *Actinomarinicola*, *Bacillariophyta* and *Oscillochloris*) affected some soil physicochemical parameters (e.g., organic matter and pH) of the rhizosphere soil and enhanced the TF of metals. Functional prediction analysis of soil bacterial community showed that the relative abundances of genes related to the synthesis of some proteins (e.g., manganese/zinc-transporting P-type ATPase C, nickel transport protein and 1-aminocyclopropane-1-carboxylate deaminase) was positively correlated with the phytoextraction/phytostabilization capacity of plants for heavy metals. This study provided theoretical guidance on selecting appropriate plants for different metal remediation applications. We also found some rhizosphere bacteria might enhance the phytoremediation of multi-metals, which could provide a reference for subsequent research.

## 1 Introduction

Due to the continuous mining activities, heavy metals (HMs) contamination and vegetation destruction are severely increasing. It is a severe threat to ecological biodiversity and human health. Therefore, soil restoration and governance need urgently be solved (Xiang et al., 2021; Zerizghi et al., 2022). The primary remediation methods for metal-contaminated soils include physical, chemical and biological strategies.

In general, physical and chemical remediation methods are costly, unfriendly to the environment, and can easily lead to secondary pollution, so that many researchers now focus on bioremediation strategies (González Henao and Ghneim-Herrera, 2021; Azhar et al., 2022). Phytoremediation has been widely used due to its advantages of being cost-effective, economical, eco-friendly, and sustainable (Ahemad, 2019; Shrivastava et al., 2019). Currently, As hyperaccumulators such as *Pteris vittata* and *Pteris cretica*, Zn hyperaccumulator *Sedum alfredii*, Cd hyperaccumulator *Viola baoshanensis*, and Mn hyperaccumulator *Phytolacca acinose* have been found in China for the phytoremediation of mine tailings (Niu et al., 2021). The effectiveness of phytoremediation depends on the chemical and physical properties of the plant, the bioavailability of metals in the soil, and the ability of rhizosphere soil microorganisms to absorb, transfer and detoxify metals (Subpiramaniyam, 2021). The removal of HMs by microorganisms has the advantages of easy use, low cost, large adsorption capacity, and high efficiency. Among these, bacteria, fungi and algae are widely used (Yin et al., 2019). In general, heavy metal ions can be adsorbed and combined by some functional groups of bacterial polysaccharide mucus layer, e.g., carboxyl, amino, phosphate, and sulfate (Yue et al., 2015). The adsorbed heavy metal ions can enter the microbial cells by metal-related enzymes or proteins, changing the redox state of heavy metal ions and thereby reducing their toxicity.

In practical applications, plants and microorganisms for bioremediation are more efficient (Vaid et al., 2022). Usually, microorganisms can improve plant extraction by increasing the availability of HMs in plants and increasing plant biomass (Wood et al., 2016; Mishra et al., 2017). Previous studies have shown that *Patescibacteria* increased the availability of HMs in the rhizosphere and promoted the remediation of heavy metal-contaminated soil by *Sedum alfredii* (Tian et al., 2022). Moreover, the plant preference for some HMs is influenced by the rhizosphere ecological characteristics, including the specific hormones, root exudates, soil nutrients, soil properties and rhizosphere soil microbes (Bais et al., 2006). The phytoremediation efficiency was regulated by soil enzyme activity and beneficial rhizosphere-associated microorganisms *Trifolium repens* L. (Lin et al., 2021). Therefore, the combined plant-microbes method can improve the heavy metal resistance of plants and achieve an ideal remediation effect. In recent years, it has also been found the limited efficiency of phytoremediation with a single plant (Buscaroli et al., 2017; Rizwan et al., 2019; Hosseinniaee et al., 2022) and the co-planting pattern of complementary plants for metals enrichment may be more efficient. The co-plantation of *Solanum nigrum* with *Quercus nuttallii* or *Quercus pagoda* effectively improved the enrichment of cadmium (>40%) and zinc (>30%) (Qu et al., 2021).

The environmental problem is severe in the lead-zinc mining area in Huayuan County, Hunan Province, China. This area is a super large ore deposit with many tailings ponds. Wastewater and ore sand was discharged through tunnels and village ditches, accumulating in the surrounding crops and villagers. Barbaric mining and poor management caused environmental pollution, damaged mine landforms, reduced vegetation coverage, soil erosion, and other problems. Therefore, we aim to explore the relationships among metals uptake of the dominant plants, rhizosphere bacterial community and the soil environment under the long-term HMs stress, analyze the ecological characteristics of the rhizosphere (physicochemical properties, soil enzyme activities and bacterial community structure), and the major microbial groups and metabolisms responsible for the oxidoreduction/detoxification/tolerance of HMs in the rhizosphere and absorption/translocation of HMs of plants.

## 2 Materials and methods

### 2.1 Sample collection and pretreatment

The Pb-Zn mining area was located in Huayuan County, Hunan Province (China), with a subtropical monsoon climate with an average temperature of 16.0°C and an average rainfall of 1363.8 mm. Environmental conditions and vegetation patterns were studied through field sampling. The vegetation pattern and distribution at different study sites were analyzed by quadratic analysis to identify dominant plant species, including *Artemisia argyi* (LA, 109°22′31.31″ E and 28°31′59.47″ N), *Boehmeria nivea* (LZ, 109°22′31.31″ E and 28°31′41.25″ N) and *Miscanthus floridulus* (LM, 109°21′41.12″ E and 28°30′46.47″ N) (**Figure S1**). There were eight replicates for each plant and the corresponding rhizospheric soil. Soil samples were collected from rhizosphere soil (5-20 cm). All soil and plant samples were sealed into clean polyethylene bags and transported to the laboratory at a low temperature (<5°C ). Fresh plants were rinsed with tap water and deionized water. Then the plant samples were dried at a high temperature of 105°C for 30 minutes and then dried at 65°C to constant weight. The dried plant tissue was then separated into roots and shoots, ground to a fine powder, and placed in polyethylene bags for further analysis. Soil samples were air-dried, ground to a particle size of less than 0.147 mm (Xiao et al., 2018), and stored in polyethylene bags until analysis. The remained soil samples were stored at -80 °C for microbial analysis.

### 2.2 Measurements of HMs in plant tissues and soil properties

Plant samples (0.5 g) were digested with 10 mL mixed acid (HNO_3_: HClO_4_, vol/vol, 4:1). The contents of HMs were measured by inductively coupled plasma atomic emission spectrometry (ICAP 7200, Thermo Fisher Scientific, England) (Li G. et al., 2020).

Soil pH was measured by a pH meter (PHS-25, Shanghai Yidi Scientific Instrument Co., Ltd., China). 5.0 g soil was dried at 65°C to constant weight to measure the moisture content (MC). Soil-available phosphorus (AP), soil-available potassium (AK), soil acid phosphatase (S.ACP), soil catalase (S.CAT) and soil peroxidase (S.POD) were measured with the test kit (Shanghai zcibio technology co., Ltd. China). Analysis of soil organic matter (OM) was measured as described by Walz et al. (Walz et al., 2017). Total nitrogen was determined using the Kjeldahl method (Bremner, 2009). Soil HMs were determined regarding the method used by Sungur et al. (Sungur et al., 2020).

### 2.3 DNA extraction and Illumina MiSeq sequencing

DNA extraction and Illumina MiSeq sequencing of soil microorganisms were performed by Shenzhen E-Gene Technology Co., Ltd., using a 16S rRNA high throughput sequencing technique. Briefly, PCR was performed using primer pair 515 F (50- GTGCCAGCMGCCGCGGTAA-30) and 806 R (50-GGACTACHVGGGTWTCTAAT-30). PCR reaction conditions were as follows: 3 min of denaturation at 95 °C , 27 cycles of 30 s at 95 °C , 30 s for annealing at 55 °C , and 45 s for elongation at 72 °C , and a final extension at 72 °C for 10 min. The raw data were deposited in the Sequence Read Archive of the National Center for Biotechnology Information database (accession number: PRJNA904575).

### 2.4 Molecular ecological network

#### 2.4.1 Network construction

In this study, the relative abundances of bacterial OTUs in the soil were used to construct phylogenetic molecular ecological networks (PMEN) using a network approach based on random matrix theory (RMT). This method can automatically identify keystone OTUs and determine the topological properties of the network. The detailed operation methods were referred to the previous study by Deng et al. ( 2012). In our study, the OTUs detected in equal or more than 3 of the 8 biological replicates were kept for network construction. The default similarity threshold is 0.98. Finally, the network interactions were visualized using Cytoscape 3.9.1 and Gephi 0.9.5.

#### 2.4.2 Module detection and keystone microorganism identification

Molecular ecological networks consist of many OTUs/genes (nodes) and interactions (links) (Dunne et al., 2008). A module is a group of nodes that have similar functions and effects. Maximum modularity can separate the OTU into multiple dense subgraphs (Tao et al., 2018). Within a module, the role of a node is characterized by its intra-module connectivity (Zi) and inter-module connectivity (Pi). Based on Zi and Pi, nodes in the network could be classified into four different roles: peripheral nodes (Zi ≤ 2.5 and Pi ≤ 0.62, nodes have only a few links with other nodes within their modules); connectors (Zi ≤ 2.5 and Pi > 0.62, nodes highly connected to several modules); module hubs (Zi > 2.5 and Pi ≤ 0.62, nodes highly connected to many nodes within their own modules); and network hubs (Zi > 2.5 and Pi > 0.62, nodes act as both module hubs and connectors (Olesen et al., 2007). Nodes playing the roles of connectors, module hubs, and network hubs might act as keystone taxa to maintain the network structure and function (Faust and Raes, 2012).

### 2.5 Functional profiling

Before functional gene prediction using PICRUSt (phylogenetic investigation of communities by reconstruction of unobserved states) described by Langille et al. (2013), the detected OTUs were reclassified using the GREENGENES reference database. Subsequently, PICRUSt used 16S rRNA genes to infer metagenome gene functional content from phylogenetic information. The predictions were precalculated for genes in databases, including the Kyoto Encyclopaedia of Genes and Genomes (KEGG). The input data were first normalized by copy number by dividing each OTU by the known 16S copy number abundance before metagenome predictions and subsequent collapse into different functional pathways. The output of PICRUSt consisted of a table of functional gene counts as KEGG orthologs (KOs). The Nearest Sequenced Taxon Index (NSTI) value was used to validate the reliability of predicted metagenomes and functional pathways.

### 2.6 Statistical analysis

A completely randomized experimental design with eight replicates per treatment was used. Statistical analysis was conducted using analysis of variance (Markowitz et al., 2014). Multiple comparison analyses among treatments were performed using IBM SPSS Statistics 26 with Tukey’s test. The translocation factors (TF) and bioconcentration factors (BCF) of three plants were calculated to evaluate the restoration potential of plants. TF evaluates the ability of plants to transport and enrich HMs from underground to aboveground. BCF reflects the ability of plants to absorb and transfer HMs into plant tissues. BCF and TF were calculated as follows: BCF= C plant/C soil; TF= C shoot/C root.

The Shannon and Simpson diversity index analysis showed species abundance and evenness. The Chord diagram of the species relationship map was used to analyze the species composition of each sample in the microbial community. A Venn diagram was used to show the general relationship between the different treatments. It was commonly used to visualize common or unique information from multiple samples. Ternary phase diagrams showed the relative abundance of species in different groups. Principal Component Analysis (PCA) and Non-metric multidimensional scaling (NMDS) analysis were used to reflect the distances of the bacterial communities among samples. LEfse analysis (LDA Effect Size analysis) was conducted to find the species with significant differences in abundance between groups. Correlation analysis was conducted to show the Spearman correlation coefficient among environmental variables and the Mantel correlation coefficient among microbial species, predicting functional genes, environmental variables and BCF/TF. All the above mapping analysis was conducted using Origin 64 and Rstudio 4.2.0.

## 3 Results

### 3.1 The contents of HMs in the shoots and roots.

The contents of HMs in the shoots and roots of plants were shown in **Table 1**. For the shoot, the content of Pb in the LA group reached 144.43 mg/kg, significantly (*p <* 0.05) higher than that in the LZ and LM groups. The content of Zn (635.81 mg/kg), Cu (81.29 mg/kg), Fe (5529.07 mg/kg) and Mn (501.26 mg/kg) in the LZ group was significantly (*p <* 0.05) higher than that in the LA group. The content of Cr (654.82 mg/kg) and Ni (90.8676 mg/kg) in the LM group was significantly (*p <* 0.05) higher than that in the LA and LZ groups. Except for Mn, the contents of other HMs in the roots of LA were significantly (*p* < 0.05) higher than those of LZ and LM in the root. Among them, the contents of Zn and Pb reached 1053.40 mg/kg and 1265.88 mg/kg, respectively.

**Table 1:** The contents of different heavy metals in three plants.

Parameter	HMs(mg/kg)	LA	LZ	LM
Shoot	As	3.28 ± 0.86 a	5.09 ± 2.64 a	5.12 ± 2.77 a
	Cd	5.3 ± 3.3 a	3.61 ± 2.75 ab	2.04 ± 0.72 b
	Cr	503.16 ± 11.33 b	549.47 ± 28.09 b	654.82 ± 82.22 a
	Cu	1063986 60.72 ± 2.59 b	81.29 ± 10.75 a	69.38 ± 25.26 ab
	Fe	2032.94 ± 864.74 b	5529.07 ± 2380.7 a	2698.95 ± 1317.88 b
	Mn	219.84 ± 77.14 b	501.26 ± 117.42 a	401.84 ± 63.64 a
	Ni	11.91 ± 3.69 b	32.98 ± 9.96 b	90.87 ± 37.45 a
	Pb	144.43 ± 56.53 a	37.06 ± 16.33 b	61.29 ± 26.96 b
	Sb	7.17 ± 1.84 a	8.46 ± 2.59 a	10.88 ± 4.57 a
	Zn	317.7 ± 43.28 b	635.81 ± 371.61 a	371.9 ± 68.1 ab
Root	As	9.86 ± 5.26 a	2.01 ± 1.37 b	2.01 ± 0.88 b
	Cd	4.56 ± 2.01 a	1.04 ± 0.88 b	1.74 ± 1.05 b
	Cr	598.18 ± 56.12 a	354.78 ± 29.29 b	355.63 ± 18.32 b
	Cu	85.04 ± 15.39 a	45.13 ± 6.81 b	40.82 ± 5.21 b
	Fe	15352.6 ± 8704.41 a	4166.79 ± 1767.31 b	3620.98 ± 1272.64 b
	Mn	12.66 ± 4.35 b	20.37 ± 5.87 b	51.11 ± 30.5 a
	Ni	58.09 ± 24.07 a	26.51 ± 18.31 b	27.41 ± 8.81 b
	Pb	1265.88 ± 410.09 a	32.67 ± 13.28 b	100.18 ± 51.92 b
	Sb	8.64 ± 3.94 a	3.41 ± 1.08 b	4.95 ± 0.95 b
	Zn	1053.39 ± 410.54 a	255.99 ± 96.69 b	360.55 ± 181.34 b

Values in the table represent mean ± standard deviation, and different lowercase letters in the table represent significant differences.

### 3.2 Soil physicochemical/biochemical properties

The physicochemical properties in the rhizosphere soil were shown in **Table 2**. The MC (2.75%-15.88%), pH (4.89-6.37), OM (3.47-21.55) and TN (0.36-2.29) were different in rhizosphere soils of different plants, and they were significantly (*p* < 0.05) higher in the LZ group than those of LM. The soil AK contents showed no significant (*p* > 0.05) difference among the three groups, but the soil AP content in the LZ and LM groups was significantly (*p* < 0.05) higher than that of LA by 18.77% and 22.53%, respectively. The activity of S-ACP in the LA and LZ groups were significantly (*p* < 0.05) higher than that in the LM group, but the activities of S-CAT and S-POD showed no significant (*p* > 0.05) differences.

**Table 2:** Physiological and biochemical indexes of soil.

Parameter	LA	LZ	LM
MC (%)	15.88 ± 1.44 a	15.35 ± 1.52 a	2.75 ± 1.12 b
pH	5.52 ± 0.85 ab	6.37 ± 0.6 a	4.89 ± 0.83 b
OM (g/kg)	6.42 ± 3.01 b	21.55 ± 2.22 a	3.47 ± 1.32 c
TN (g/kg)	0.63 ± 0.21 b	2.29 ± 0.91 a	0.36 ± 0.15 b
AP (μmol/g)	0.28 ± 0.02 b	0.33 ± 0.05 a	0.34 ± 0.03 a
AK (mg/kg)	10.85 ± 0.2 a	10.78 ± 0.13 a	10.8 ± 0.14 a
As (mg/kg)	11.17 ± 3.94 b	16.51 ± 2.42 b	34.41 ± 10.44 a
Cd (mg/kg)	2.53 ± 1.08 c	7.42 ± 0.52 a	4.03 ± 1.2 b
Cr (mg/kg)	3.83 ± 6.95 b	44.06 ± 12.25 a	40.77 ± 20.47 a
Cu (mg/kg)	29.32 ± 13.64 c	93.68 ± 11.55 a	71.18 ± 10.62 b
Fe (mg/kg)	14148.06 ± 4733.69 b	45100.6 ± 1821 a	40984.16 ± 10974.14 a
Mn (mg/kg)	761.88 ± 300.12 c	2321.23 ± 360.87 a	1368.95 ± 320.17 b
Ni (mg/kg)	21.69 ± 5.29 b	45.67 ± 1.15 a	49.54 ± 13.21 a
Pb (mg/kg)	1130.67 ± 492.75 a	392.18 ± 32.72 b	807.54 ± 250.25 a
Sb (mg/kg)	6.24 ± 2.04 a	3.6 ± 0.97 b	3.9 ± 1.69 b
Zn (mg/kg)	917.67 ± 303.19 b	1351.12 ± 53.11 a	796.31 ± 296.53 b
S-ACP (nmol/d·g)	6000.73 ± 345.23 a	6788.41 ± 512.17 a	4351.27 ± 954.43 b
S-CAT (U/g)	110.69 ± 0.68 a	109.74 ± 0.96 a	110.86 ± 1.15 a
S-POD (μmol/d/g)	401.24 ± 200.31 a	430.33 ± 174.42 a	350.84 ± 97.79 a

Values in the table represent mean ± standard deviation, and different lowercase letters in the table represent significant differences.

In addition, the contents of As (11.17 mg/kg), Cd (2.53 mg/kg), Cr (3.83 mg/kg), Cu (29.32 mg/kg), Fe (14148.06 mg/kg), Mn (761.88 mg/kg), and Ni (21.69 mg/kg) in the LA group were significantly (*p* < 0.05) lower than those in the LZ or LM group. The Pb content in the LZ group was 392.18 mg/kg, which was significantly (*p* < 0.05) lower than that of LA and LM.

### 3.3 Translocation factor and Bioconcentration factor

The results of BCF and TF were shown in **Table 3**. The BCFs of Cd (2.31), Cr (105.40), Cu (2.93) and Ni (1.66) were greater than 1 in the LA group, while only the BCFs of Cr (11.04) and Sb (1.74) in the LZ group and the BCF of Cr (16.36) and Sb (3.08) in the LM group were greater than 1. In addition, LZ also has strong TF (TF > 1) for all other metals, LM has strong TF (TF > 1) for all metals except Mn and Sb, and LA has strong TF (TF > 1) for Cd and Zn. The TFs of Cr, Cu, and Ni in LA also reached 0.84, 0.71, and 0.83, which were relatively higher than the TFs of other HMs in the same plant species. It was found that the TF of LZ for Cd, Cu, Mn, Ni, Pb, Sb, and Zn were higher than those of LA and LM, while the TF of LM for As, and Cr, was the strongest.

**Table 3:** Translocation factors and Bioconcentration factors.

	Treatment	As	Cd	Cr	Cu	Fe	Mn	Ni	Pb	Sb	Zn
TF	LA	0.33	**1.16**	0.84	0.71	0.21	0.11	0.83	0.3	0.13	**17.36**
	LZ	**2.53**	**3.47**	**1.55**	**1.8**	**1.24**	**1.13**	**2.48**	**2.48**	**1.33**	**24.61**
	LM	**2.54**	**1.17**	**1.84**	**1.7**	**3.31**	0.61	**2.2**	**1.03**	0.75	**7.86**
BCF	LA	0.64	**2.31**	**105.4**	**2.93**	0.72	0.19	**1.66**	0.9	**1.4**	0.76
	LZ	0.23	0.32	**11.04**	0.68	0.11	0.12	0.65	0.09	**1.74**	0.33
	LM	0.12	0.51	**16.36**	0.79	0.09	0.18	**1.35**	0.1	**3.08**	0.51

Bold font means that BCF is greater than 1 or TF is greater than 1.

### 3.4 Overview of bacterial community in the rhizosphere soil

**Figure 1:**
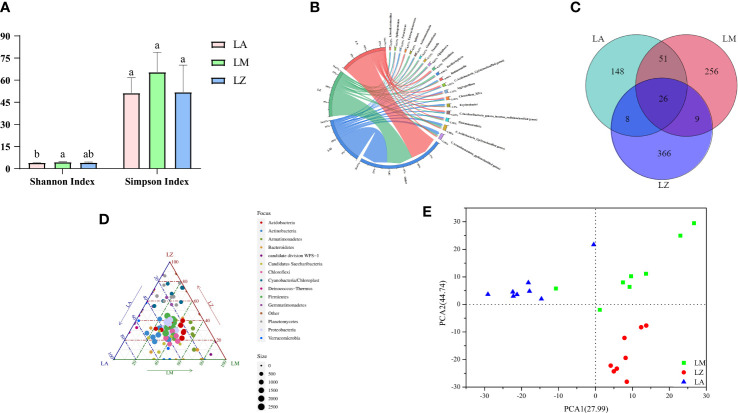
Structure, diversity and composition of rhizospheric soil bacterial communities in three dominant plants. **(A)** Shannon Index and Simpson Index; **(B)** The abundance of microorganisms at the genus level; **(C)** Venn diagram; **(D)** The ternary map of microorganisms at the phylum level; **(E)** Principal Component Analysis. In the ternary phase diagram, different points represent different species, the size of the points represents the average abundance of the species in different groups, the gray points represent the non-enrichment, and the points of each color respectively represent the enriched groups.

Simpson Index showed no significant (*p* > 0.05) differences among the three groups, but the Simpson Index in the LM group reached 65.51, which was higher than the other two groups. The Shannon Index showed that the microbial diversity of LA, LM, and LZ were 4.11, 4.18, and 4.44, respectively. Among them, LM had the most abundant diversity and was significantly higher than LA (**Figure 1A**). According to the Venn diagram, there were 26 duplicated OTUs in 864 OTUs (**Figure 1C**). The abundance chord chart showed that soil bacterial communities mainly consisted of *Armatimonadetes_gp4*, *Gp3*, and *Thermanaerothrix*, and the dominant genera were different among three groups (**Figure 1B**): they were *Ornatilinea* (4.47%), *Gp16* (3.63%), and *Saccharibacteria_genera_incertae_sedis* (3.60%) in the LA group; they were *Armatimonadetes_gp4* (6.92%), *Gp3* (4.93%), and *Thermanaerothrix* (3.43%) in the LM group; They were *Gp3* (4.66%), *Bacillariophyta* (4.30%), and *Iphinoe* (3.50%) in the LZ group. A ternary plot analysis performed on the phylum level showed that Actinobacteria, Cyanobacteria/Chloroplast, Chloroflexi, Firmicutes, and Proteobacteria were the dominant phyla (**Figure 1D**). Armatimonadetes dominated LM, and Planctomycetes dominated LZ. Results of PCA showed that points of samples in the three groups separated from each other group (**Figure 1E**), and similar results were obtained by dissimilarity tests, suggesting that significant (*p* < 0.05) differences occurred in the rhizosphere bacterial community structure of different plant (**Table S1**).

**Figure 2:**
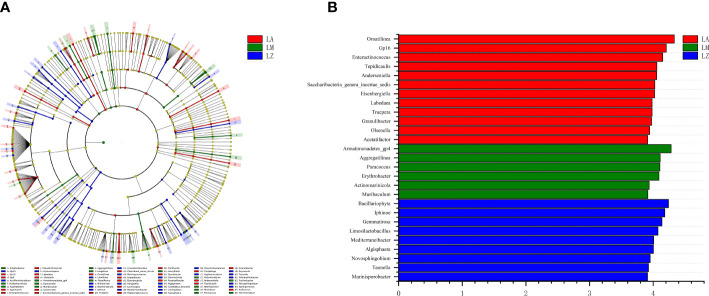
Linear discriminant analysis effect size (LEfSe). **(A)** The evolutionary branching diagram, and **(B)** the distribution histogram.

Further LEfse analysis at the genus level was shown in **Figure 2**. The cladogram (**Figure 2A**) compares the differences of genera between three plants, and LDA analysis (**Figure 2B**) shows the main genera with significant (*p* < 0.05) differences. The relative abundances of genera (e.g., *Ornatilinea*, *Gp16*, *Enteractinococcus*) were highest in the LA group; The relative abundances of genera (e.g., *Armatimonadetes_gp4*, *Aggregatilinea*, *Paracoccus*) were highest in the LM group; The relative abundances of genera (e.g., *Bacillariophyta*, *Iphinoe*, *Gemmatirosa*) were highest in the LZ group.

### 3.5 Co-occurrence networks analysis

**Figure 3:**
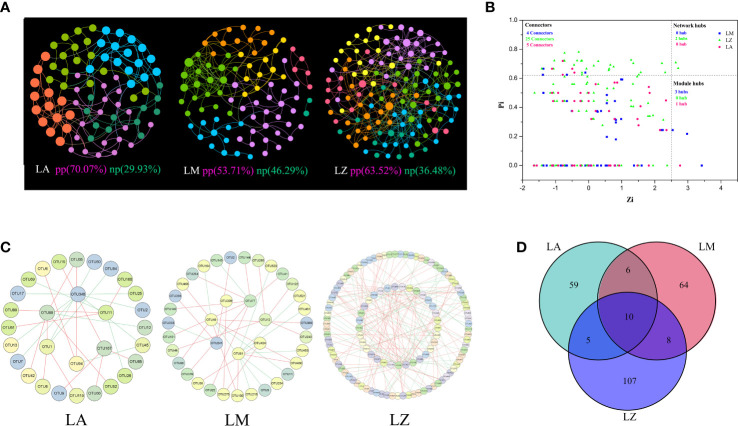
Co-occurrence Networks analysis. **(A)** Co-occurrence network; **(B)** ZP diagram; **(C)** Sub-network analysis diagram; **(D)** Venn diagram of key nodes. Different colors represent different modules, and the key nodes indicated Zi>2.5 or Pi>0.62.

Microbial population data were analyzed using the RMT-based network approach to discern the ecological network. Three networks were constructed based on the 16S rRNA gene sequencing data of three plans, respectively (**Figure 3A**). Major topological properties of the empirical MENs of microbial communities in the eight groups are shown in **Table S2**. With the same threshold (0.980), their correlation values were more than 0.580, indicating that the degree of distributions in the constructed molecular ecological networks fits the power law model well. There were 134 links and 67 nodes in the LA group, of which the positive links accounted for 70.07%, and the negative links accounted for 29.93%. There were 137 links and 80 nodes in the LM group, of which the positive links accounted for 53.71%, and the negative links accounted for 46.29%. There were 175 links and 88 nodes in the LZ group, of which the positive links accounted for 63.52% and the negative links accounted for 36.48% (**Figure 3A**). The network indexes showed that the microbial network of LZ was the most complex, but the number of modules in the LM group was higher than that of LA and LZ (**Table S2**). Results of ZP analysis showed that there were 6, 7 and 27 key OTUs in the LA, LM and LZ groups (**Figure 3B**). Sub-network analysis of key OTUs revealed that OTU167 (*Geothermobacterium*, 4 connections, 4 positive correlations) and OTU89 (*Clostridium XlVa*, 5 connections, 4 positive correlations) were the core OTUs in the LA group. OTU77 (*Paracoccus*, 8 connections, 7 positive correlations) and OTU12 (*Algisphaera*, 4 connections, 4 positive correlations) were the core OTUs in the LM group. The core OTUs in the LZ group were OTU57 (*Iphinoe*, 19 connections, 16 positive correlations), OTU61 (*Paracoccus*, 18 connections, 14 positive correlations), OTU268 (*Pseudoscardovia*, 6 connections, 6 positive correlations), OTU111 (*Limosilactobacillus*, 7 connections, 6 positive correlations) (**Figure 3C**). Venn diagram analysis of essential microorganisms showed that 10 key OTUs were shared by LA, LM, and LZ (**Figure 3D**), which belonged to the genera, including *Armatimonadetes_gp4*, *Gp3*, *Algisphaera*, *Clostridium XlVa*, *Robinsoniella*, *Taonella*, *Verrucosispora*, *Erythrobacter*, and *Thermanaerothrix*.

### 3.6 Correlation analysis

**Figure 4:**
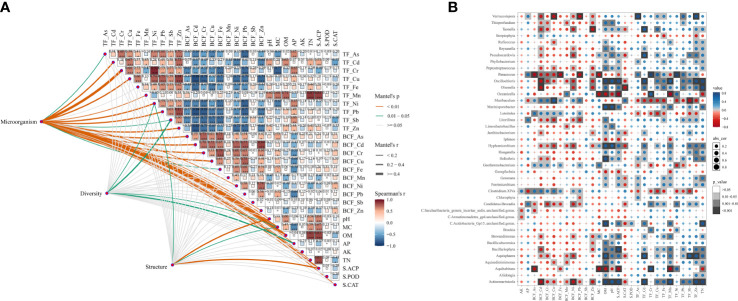
Correlation analysis among bacterial communities, TFs and BCFs of heavy metals, and soil physicochemical properties. **(A)** The relationships among the different parameters (microorganism, diversity, structure), HMs TFs, BCFs and soil environmental parameters by Mantel tests; **(B)** Spearman correlations of genus and soil environmental factors.

According to the correlation analysis (**Figure 4A**), it was shown that the key microorganisms were mainly related to the BCF of Cu, Cr, Cd, Fe, Ni and Zn, and the TF of As, Cd, Cr, Cu, Mn, Ni, Pb and Zn. S.ACP, S.CAT, pH, MC, OM, AP and TN were also significantly (*p <* 0.05) correlated to the key microorganisms. The diversity of the bacterial community was mainly related to the TF of Ni and Sb, as well as MC and AP. Bacterial community structures were significantly (*p <* 0.05) correlated to the TF of Ni, the activity of S.ACP, soil pH, and the contents of MC. Most HMs were mainly related to the contents of AP and MC in soil (**Figure 4A**). TF_Cd was significantly (*p <* 0.05) positively correlated to the contents of OM/TN, soil pH, and the activity of S.ACP. TF_Fe was significantly (*p <* 0.05) positively correlated with TN, BCF_Mn, and BCF_Ni were significantly (*p <* 0.05) negatively correlated with the content of OM (*p <* 0.05). There was no significant (*p >* 0.05) correlation between BCF_Sb and soil physicochemical properties. Similarly, there was no significant (*p >* 0.05) difference between the TF and BCF of HMs and the soil contents of AK. Further correlation tests explored the relationships among microorganisms, HMs, and physiochemical properties in soil. It was found that the essential microorganisms were mainly negatively correlated with S.CAT, positively correlated with the TF of HMs, and had no significant (*p >* 0.05) correlation with S.POD. Interestingly, the Bacteroidetes was significantly (*p <* 0.05) negatively associated with soil nutrients. Furthermore, most microbiotas were negatively correlated with the BCF of HMs. The correlations between microorganisms and environmental factors/BCF/TF were shown in **Figure 4B**. Some microorganisms (e.g., *Actinomarinicola*, *Bacillariophyta* and *Oscillochloris*) were positively related to organic matter, pH and the TF of metals, while were negatively related to the BCF of metals.

### 3.7 Analysis of predictive functional genes

**Figure 5:**
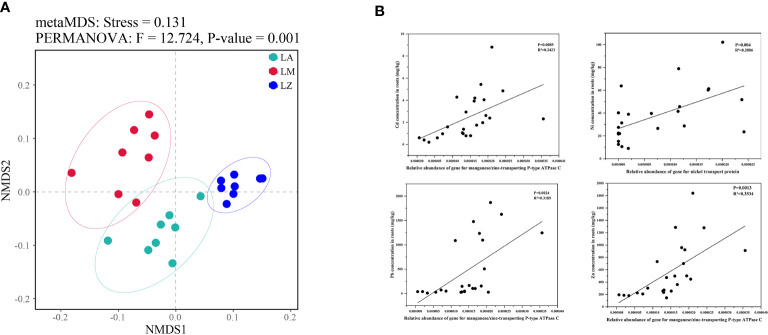
NMDS analysis of the structure in predicting genes **(A)** and the correlations between some genes and the contents of heavy metals in plant roots **(B)**.

NMDS analysis showed significant inter-group differences in the predictive functional genes contained in soil microorganisms from different plant rhizosphere, while intra-group differences were slight (**Figure 5A**). There were significant (*p <* 0.05) differences in the relative abundances of some functional genes in different microbial communities (**Table S3**). The relative abundances of the genes about 1-aminocyclopropane-1-carboxylate deaminase (ACC deaminase), tryptophan synthase alpha/beta chain, nickel transport protein and manganese/Zinc-transporting P-type ATPase C were the most abundant in LA. The relative abundances of the genes about nonribosomal peptide synthetases were the highest in LZ (**Table 4**). Furthermore, some genes were further selected to make fitting curves with the contents of HMs in plant root tissues (**Figure 5B**). The relative abundance of the gene about manganese/zinc-transporting P-type ATPase C was significantly (*p <* 0.01) positively correlated with the contents of Cd, Zn, and Pb, and the relative abundance of the gene about nickel transport protein was significantly (*p <* 0.01) positively correlated with the contents of Ni.

**Table 4:** Relative abundance of key functional genes in different rhizosphere microbial communities.

Genes for proteins/enzymes	LA	LM	LZ
ACC deaminase	7.96E-05a	5.35E-05b	7.53E-05a
Tryptophan synthase alpha/beta chain	6.71E-04a	5.82E-04b	4.89E-04c
Nonribosomal peptide synthetases	8.82E-04b	7.33E-04b	1.21E-03a
Nickel transport protein	1.72E-05a	3.72E-06b	3.47E-07b
Manganese/Zinc-transporting P-type ATPase C	2.06E-05a	7.48E-06b	1.51E-05a

## 4 Discussion

**Figure 6:**
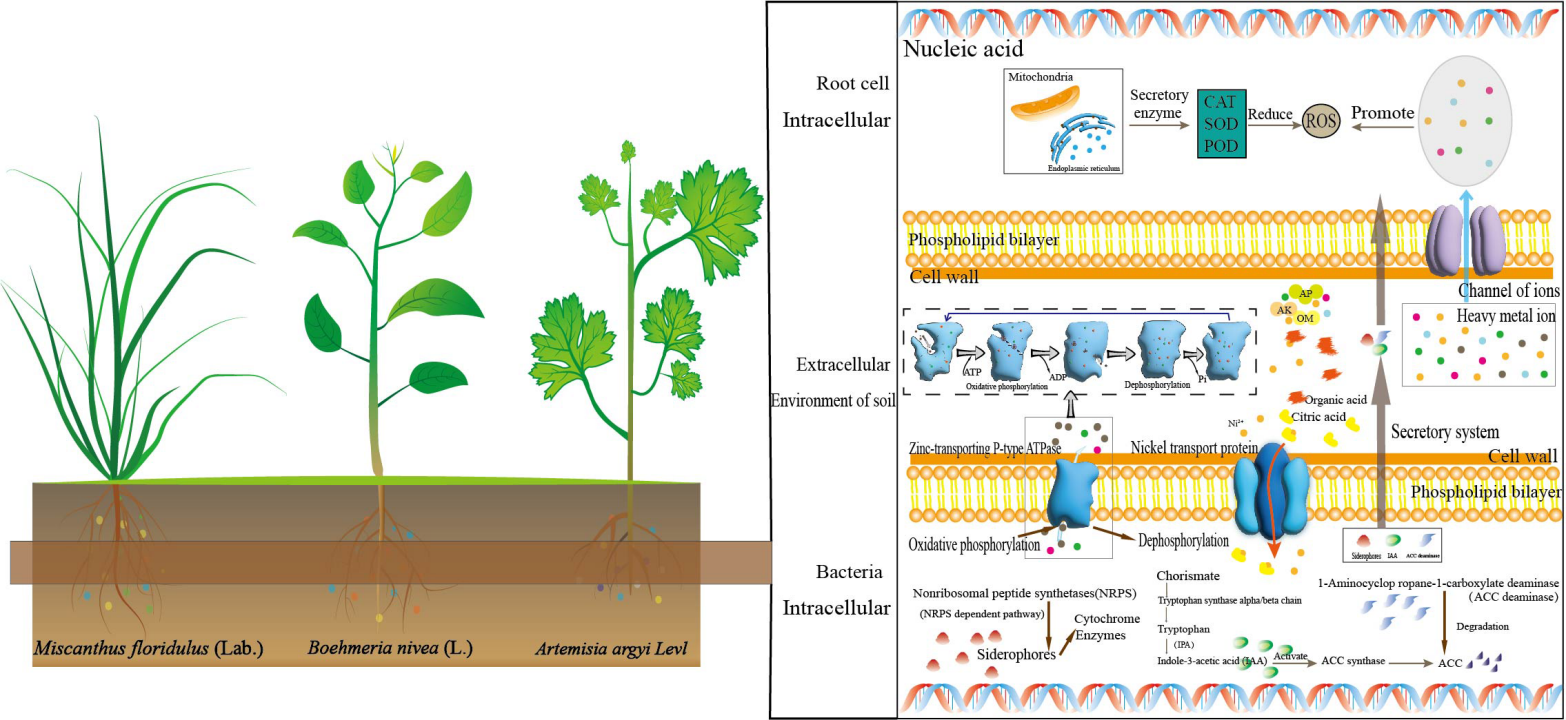
Schematic plot of the interaction between rhizosphere bacteria and plants in multi-HMs contaminated soil.

In recent decades, our demand for metal resources has been increasing, which accelerated the mining of metal mines. The continuous expansion of mining areas led to more and more arable land facing environmental pollution (Diaz-Morales et al., 2021; Li et al., 2022). The increase in HMs will stimulate the production of a large number of reactive oxygen species, which will seriously impact the quality of crops and vegetables (Clemens and Ma, 2016) and lead to human health risks (Xiang et al., 2021). Phytoremediation is a sustainable approach to remediating contaminated sites (Hasnaoui et al., 2020). Thus, the study of the interaction between the hyperaccumulators and microorganisms can help formulate effective remediation strategies for mining areas (Xuan et al., 2021). In this study, we investigated the rhizosphere ecological characteristics of the dominant plants around the mining area and further explored their potential for multi-metal(loid)s phytoremediation. It was found that As, Cd, Cu, Pb and Zn have reached potentially hazardous levels (**Table 1**). We found that *Artemisia argyi, Miscanthus floridulus*, *Boehmeria nivea* are the dominant enrichment plants in this area, and the application of dominant plants combined with microbial communities for the bioremediation of soil pollution was explored (**Figure 6**).

Microorganisms can directly or indirectly change the availability of metals and promote plant growth and absorption of HMs by regulating soil physicochemical properties or secretion of secondary metabolites. Our study found that Cyanobacteria/Chloroplast, Chloroflexi and Acidobacteria were key phyla for the plant to accumulate HMs. Different plants had different dominant rhizosphere flora, which affected the remediation of different HMs. The dominant phylum Cyanobacteria/Chloroplast in LZ was significantly (*p<*0.05) positively correlated with TF of Cd, Cu, Mn, Pb and Zn (**Figure S2**). However, few studies reported the responses of Cyanobacteria/Chloroplast and their roles in phytoremediation under HMs stress, and it might need further study to verify. Chloroflexi was the dominant phylum in the LA group (**Figure 1B**), significantly (*p <* 0.05) higher than in LM and LZ (**Figure 2B**). Studies have found that Chloroflexi had an obvious advantage in polluted soil (Hemmat-Jou et al., 2018; Koner et al., 2022), which could rapidly adapt to heavy metal stress and affect the content of organic matter to control the practical availability of Cr and Pb for plants (Tang et al., 2019). It is consistent with this study, where Chloroflexi was also significantly (*p <* 0.05) positively correlated with the organic matter (**Figure S2**). Acidobacteria was the dominant phylum in the LM group (**Figure 1B**) and was significantly (*p <* 0.05) positively correlated with BCF of Cr (**Figure S2**). Previous research found that the relative abundance of Acidobacteria was inversely related to pH (Debnath et al., 2016). Sun et al. (2022) found that the relative abundance of Acidobacteria was positively correlated to the contents of total Cr and available Cr, and Acidobacteria could cut down soil pH. The key genera of LA were *Truepera* and *Anderseniella*, that of LM were *Paracoccus* and *Erythrobacter*, and of LZ was *Novosphingobium*. The oxidation-reduction and nitrogen fixation activities of *Truepera* allow it to support plant growth (Zhou et al., 2022). *Anderseniella* can eliminate metabolic wastes, heavy metals, and aromatic chemicals (Karimi et al., 2019). *Paracoccus* can produce acid, alter the chemical and physical parameters in the soil and encourage plants to absorb heavy metals (Carvalho et al., 2018). *Erythrobacter* is an iron metabolism bacterium that can secrete iron carriers and promote plant growth (Li L. et al., 2020). *Novosphingobium* has strong antioxidant activity and can slow down the toxic effect of heavy metals on plants (Petruk et al., 2018).

Except for the dominant flora of different plants, the study found that they shared the same vital microorganisms (**Figure 3D**). These microorganisms (e.g., Proteobacteria, Acidobacteria and Firmicutes) could assist plants in accumulating HMs. Studies have found that in the harsh tailings environment, the colonies mentioned above belonged to the dominant flora and could benefit the expression of metal resistance genes (Guo et al., 2017; Jiang et al., 2021; Koner et al., 2022). HMs in the soil can affect the growth of microorganisms through protein denaturation, cell membrane damage and inhibition of RNA expression and metabolism (Wang et al., 2020; Duan et al., 2022). It has also been studied that HMs stress enhanced the functional fitness of endophytic bacterial communities (Yao et al., 2022). Most microorganisms were positively correlated to the contents of soil organic matter so that we might stabilize soil microbial communities and increase the abundance of beneficial bacteria by improving soil fertility. Notably, the research found that the abundance of Bacteroidetes increased in extremely harsh soils, and Bacteroidetes transferred and enriched a large amount of Ni (**Figure S2**), which is consistent with Jiao et al. (2022) findings. Besides, Tang et al. (2019) found that Bacteroidetes also affected the availability of Cu and Zn.

Okkeri and Haltia (1999; 2006) found that the zinc-transporting P-type ATPase C was not only related to the transport of Zn but also to Cd and Pb, which is consistent with our genetic prediction. Similarly, nickel transport protein is a novel metal regulatory protein associated with heavy metal Ni transport (Dosanjh and Michel, 2006; Wang et al., 2014). In addition, there are functional genes secreting siderophore (Carroll and Moore, 2018), indoleacetic acid (Estenson et al., 2018) and ACC deaminase (Glick, 2005) in rhizosphere soil microorganisms. It is indicated that many plant growth-promoting bacteria (PGPB) resist HMs in rhizosphere soil. They significantly improved plant growth in heavy metal-contaminated soils and could enhance heavy metal phytoremediation by binding super-enriched plants (Ahemad, 2019). Xu et al. (2022) found that Actinobacteriota and Gemmatimonadota promoted plant growth and fixed Cd in rhizosphere soil. Li et al. (2022) found that *Sphingomonas* promoted plant growth and degraded organometallic compounds to remediate soil HMs contamination. Similarly, Ham et al. (2022) found that *Pseudopyroactor* promoted plant growth and improved antioxidant capacity. PGPB significantly improve plant growth in heavy metal-contaminated soils (Ahemad, 2019) and may represent potential bacterial strain resources in plant-PGPR combined remediation of HM-contaminated soils.

The presence of HMs significantly destabilizes network structure (Jiang et al., 2020; Caili et al., 2022). A more complex network represents that microbial activities and interactions are more active and intensive, which might have beneficial functions in the phytoremediation of HMs in soils (Hou et al., 2019). Plants consistently interact with a core set of microbes contributing to plant performance (Luo et al., 2022). We found that the network of LZ was the most complex (**Figure 3C**), and *Boehmeria nives* (L.) showed higher TFs of all ten metals. Thus, a good rhizosphere ecological network might be helpful for plants to absorb heavy metals.

## 5 Conclusion

According to our investigation of BCF and TF for metals, LA preferred accumulating Cd, LZ preferred accumulating Cr and Sb, and LM preferred accumulating Cr and Ni. The bioaccumulating capacity for multi-metals of *Artemisia argyi* Levl was more potent, while the translocating capacity was weaker than that of *Miscanthus floridulus* (Lab.) and *Boehmeria nives* (L.). The dominant bacterial groups, ecological networks and soil properties (e.g., available phosphorus and moisture content) were different in the rhizosphere soil of these three plants. A higher proportion of positive links in the ecological network might enhance the metal uptake of plants. PICRUSt analysis and correlation tests indicated that genes related to the synthesis of proteins (e.g., manganese/zinc-transporting P-type ATPase C, nickel transport protein and ACC deaminase) could also promote phytoremediation. The rhizosphere bacterial community contained genera, such as *Truepera*, *Anderseniella*, *Paracoccus*, *Erythrobacter* and *Novosphingobium*, which may represent potential bacterial strain resources for plant-microbes combined remediation of HM-contaminated soils.

## Data availability statement

The datasets presented in this study can be found in online repositories. The names of the repository/repositories and accession number(s) can be found in the article/**Supplementary Material**.

## Author contributions

YX, JF, and SL contributed to the study’s conception and design. Research, material preparation, data collection, and analysis were performed by LC, CL, RC, JM and GL. The first draft of the manuscript was written by LC, YX, BY commented on previous versions of the manuscript. All aforementioned authors read and approved the final manuscript. All authors contributed to the article and approved the submitted version.

## References

[B1] Ahemad M. (2019). Remediation of metalliferous soils through the heavy metal resistant plant growth promoting bacteria: Paradigms and prospects. ARAB J. Chem. 12 (7), 1365–1377. doi: 10.1016/j.arabjc.2014.11.020

[B2] Azhar U. Ahmad H. Shafqat H. Babar M. Shahzad Munir H. M. Sagir M.. (2022). Remediation techniques for elimination of heavy metal pollutants from soil: A review. Environ. Res. 214 (Pt 4), 113918. doi: 10.1016/j.envres.2022.113918 35926577

[B3] Bais H. P. Weir T. L. Perry L. G. Gilroy S. Vivanco J. M. (2006). The role of root exudates in rhizosphere interactions with plants and other organisms. Annu. Rev. Plant Biol. 57, 233–266. doi: 10.1146/annurev.arplant.57.032905.105159 16669762

[B4] Bremner J. M. (2009). Determination of nitrogen in soil by the kjeldahl method. J. AGR SCI-CAMBRIDGE. 55 (01), 11. doi: 10.1017/s0021859600021572

[B5] Buscaroli A. Zannoni D. Menichetti M. Dinelli E. (2017). Assessment of metal accumulation capacity of *Dittrichia viscosa* (L.) greuter in two different Italian mine areas for contaminated soils remediation. J. GEOCHEM. Explor. 182, 123–131. doi: 10.1016/j.gexplo.2016.10.001

[B6] Caili S. Pan W. Guanghao W. Xingjie K. (2022). Heavy metal pollution decreases the stability of microbial co-occurrence networks in the rhizosphere of native plants. Front. Environ. Sci. 1631. doi: 10.3389/fenvs.2022.979922

[B7] Carroll C. S. Moore M. M. (2018). Ironing out siderophore biosynthesis: a review of non-ribosomal peptide synthetase (NRPS)-independent siderophore synthetases. Crit. Rev. Biochem. Mol. Biol. 53 (4), 356–381. doi: 10.1080/10409238.2018.1476449 29863423

[B8] Carvalho G. Pedras I. Karst S. M. Oliveira C. S. S. Duque A. F. Nielsen P. H.. (2018). Functional redundancy ensures performance robustness in 3-stage PHA-producing mixed cultures under variable feed operation. New Biotechnol. 40, 207–217. doi: 10.1016/j.nbt.2017.08.007 28838619

[B9] Clemens S. Ma J. F. (2016). Toxic heavy metal and metalloid accumulation in crop plants and foods. Annu. Rev. Plant Biol. 67, 489–512. doi: 10.1146/annurev-arplant-043015-112301 27128467

[B10] Debnath R. Yadav A. Gupta V. K. Singh B. P. Handique P. J. Saikia R. (2016). Rhizospheric bacterial community of endemic *Rhododendron arboreum* sm. ssp. delavayi along eastern himalayan slope in tawang. Front. Environ. Sci. 07. doi: 10.3389/fpls.2016.01345 PMC5009118 27642287

[B11] Deng Y. Jiang Y.-H. Yang Y. He Z. Luo F. Zhou J. (2012). Molecular ecological network analyses. BMC Bioinf. 13 (1), 113. doi: 10.1186/1471-2105-13-113 PMC3428680 22646978

[B12] Diaz-Morales D. M. Erasmus J. H. Bosch S. Nachev M. Smit N. J. Zimmermann S.. (2021). Metal contamination and toxicity of soils and river sediments from the world’s largest platinum mining area. Environ. pollut. 286, 117284. doi: 10.1016/j.envpol.2021.117284 33984780

[B13] Dosanjh N. S. Michel S. L. (2006). Microbial nickel metalloregulation: NikRs for nickel ions. Curr. Opin. Chem. Biol. 10 (2), 123–130. doi: 10.1016/j.cbpa.2006.02.011 16504569

[B14] Duan C. Wang Y. Wang Q. Ju W. Zhang Z. Cui Y.. (2022). Microbial metabolic limitation of rhizosphere under heavy metal stress: Evidence from soil ecoenzymatic stoichiometry. Environ. pollut. 300, 118978. doi: 10.1016/j.envpol.2022.118978 35150803

[B15] Dunne J. A. Williams R. J. Martinez N. D. Wood R. A. Erwin D. H. (2008). Compilation and network analyses of cambrian food webs. PloS Biol. 6 (4), e102. doi: 10.1371/journal.pbio.0060102 18447582 PMC2689700

[B16] Estenson K. Hurst G. B. Standaert R. F. Bible A. N. Garcia D. Chourey K.. (2018). Characterization of indole-3-acetic acid biosynthesis and the effects of this phytohormone on the proteome of the plant-a microbe pantoea sp. YR343. J. Proteome Res. 17 (4), 1361–1374. doi: 10.1021/acs.jproteome.7b00708 29464956

[B17] Faust K. Raes J. (2012). Microbial interactions: from networks to models. Nat. Rev. Microbiol. 10 (8), 538–550. doi: 10.1038/nrmicro2832 22796884

[B18] Glick B. R. (2005). Modulation of plant ethylene levels by the bacterial enzyme ACC deaminase. FEMS Microbiol. Lett. 251 (1), 1–7. doi: 10.1016/j.femsle.2005.07.030 16099604

[B19] González Henao S. Ghneim-Herrera T. (2021). Heavy metals in soils and the remediation potential of bacteria associated with the plant microbiome. Front. Environ. Sci. 9. doi: 10.3389/fenvs.2021.604216

[B20] Guo H. Nasir M. Lv J. Dai Y. Gao J. (2017). Understanding the variation of microbial community in heavy metals contaminated soil using high throughput sequencing. Ecotoxicol. Environ. Saf. 144, 300–306. doi: 10.1016/j.ecoenv.2017.06.048 28645031

[B21] Ham S. H. Yoon A. R. Oh H. E. Park Y. G. (2022). Plant growth-promoting microorganism *Pseudarthrobacter* sp. NIBRBAC000502770 enhances the growth and flavonoid content of *Geum aleppicum* . Microorganisms 10 (6), 1241. doi: 10.3390/microorganisms10061241 35744759 PMC9231079

[B22] Hasnaoui S. E. Fahr M. Keller C. Levard C. Angeletti B. Chaurand P.. (2020). Screening of native plants growing on a Pb/Zn mining area in eastern Morocco: Perspectives for phytoremediation. Plants (Basel) 9 (11), 1458. doi: 10.3390/plants9111458 33137928 PMC7693513

[B23] Hemmat-Jou M. H. Safari-Sinegani A. A. Mirzaie-Asl A. Tahmourespour A. (2018). Analysis of microbial communities in heavy metals-contaminated soils using the metagenomic approach. Ecotoxicology. 27 (9), 1281–1291. doi: 10.1007/s10646-018-1981-x 30242595

[B24] Hosseinniaee S. Jafari M. Tavili A. Zare S. Cappai G. De Giudici G. (2022). Perspectives for phytoremediation capability of native plants growing on angouran Pb-zn mining complex in northwest of Iran. J. Environ. Manage 315, 115184. doi: 10.1016/j.jenvman.2022.115184 35523070

[B25] Hou J. Liu W. Wu L. Ge Y. Hu P. Li Z.. (2019). Rhodococcus sp. NSX2 modulates the phytoremediation efficiency of a trace metal-contaminated soil by reshaping the rhizosphere microbiome. Appl. Soil Ecol. 133, 62–69. doi: 10.1016/j.apsoil.2018.09.009

[B26] Jiang X. Liu W. Xu H. Cui X. Li J. Chen J.. (2021). Characterizations of heavy metal contamination, microbial community, and resistance genes in a tailing of the largest copper mine in China. Environ. pollut. 280, 116947. doi: 10.1016/j.envpol.2021.116947 33780842

[B27] Jiang R. Wang M. Chen W. Li X. Balseiro-Romero M. (2020). Changes in the integrated functional stability of microbial community under chemical stresses and the impacting factors in field soils. Ecol. Indic. 110, 105919. doi: 10.1016/j.ecolind.2019.105919

[B28] Jiao A. Gao B. Gao M. Liu X. Zhang X. Wang C.. (2022). Effect of nitrilotriacetic acid and tea saponin on the phytoremediation of Ni by Sudan grass (*Sorghum sudanense* (Piper) stapf.) in Ni-pyrene contaminated soil. Chemosphere 294, 133654. doi: 10.1016/j.chemosphere.2022.133654 35066084

[B29] Karimi E. Keller-Costa T. Slaby B. M. Cox C. J. da Rocha U. N. Hentschel U.. (2019). Genomic blueprints of sponge-prokaryote symbiosis are shared by low abundant and cultivatable *Alphaproteobacteria* . Sci. Rep. 9 (1), 1999. doi: 10.1038/s41598-019-38737-x 30760820 PMC6374434

[B30] Koner S. Tsai H.-C. Chen J.-S. Hussain B. Rajendran S. K. Hsu B.-M. (2022). Exploration of pristine plate-tectonic plains and mining exposure areas for indigenous microbial communities and its impact on the mineral-microbial geochemical weathering process in ultramafic setting. Environ. Res. 214 (Pt 2), 113802. doi: 10.1016/j.envres.2022.113802 35810813

[B31] Langille M. G. Zaneveld J. Caporaso J. G. McDonald D. Knights D. Reyes J. A.. (2013). Predictive functional profiling of microbial communities using 16S rRNA marker gene sequences. Nat. Biotechnol. 31 (9), 814–821. doi: 10.1038/nbt.2676 23975157 PMC3819121

[B32] Li L. Bai S. Li J. Wang S. Tang L. Dasgupta S. (2020). Volcanic ash inputs enhance the deep-sea seabed metal-biogeochemical cycle: A case study in the yap trench, western pacific ocean. Mar. Geol. 430, 106340. doi: 10.1016/j.margeo.2020.106340

[B33] Li G. Dongping Q. Xusong Y. Gang Y. (2020). Determination of heavy metals in the plant sample pretreatment methods. J. Phys.: Conf. Series. 1549 (2), 022008. doi: 10.1088/1742-6596/1549/2/022008

[B34] Li Q. Xiang P. Zhang T. Wu Q. Bao Z. Tu W.. (2022). The effect of phosphate mining activities on rhizosphere bacterial communities of surrounding vegetables and crops. Sci. Total Environ. 821, 153479. doi: 10.1016/j.scitotenv.2022.153479 35092784

[B35] Lin H. Liu C. Li B. Dong Y. (2021). *Trifolium repens l.* regulated phytoremediation of heavy metal contaminated soil by promoting soil enzyme activities and beneficial rhizosphere associated microorganisms. J. Hazard Mater. 402, 123829. doi: 10.1016/j.jhazmat.2020.123829 33254810

[B36] Luo J. Gu S. Guo X. Liu Y. Tao Q. Zhao H.-P.. (2022). Core microbiota in the rhizosphere of heavy metal accumulators and its contribution to plant performance. Environ. Sci. Technol. 56, 18, 12975–12987. doi: 10.1021/acs.est.1c08832 36067360

[B37] Markowitz V. M. Chen I. M. Chu K. Szeto E. Palaniappan K. Pillay M.. (2014). IMG/M 4 version of the integrated metagenome comparative analysis system. Nucleic Acids Res. 42 (Database issue), D568–D573. doi: 10.1093/nar/gkt919 24136997 PMC3964948

[B38] Mishra J. Singh R. Arora N. K. (2017). Alleviation of heavy metal stress in plants and remediation of soil by rhizosphere microorganisms. Front. Microbiol. 8. doi: 10.3389/fmicb.2017.01706 PMC5592232 28932218

[B39] Niu H. Leng Y. Li X. Yu Q. Wu H. Gong J.. (2021). Behaviors of cadmium in rhizosphere soils and its interaction with microbiome communities in phytoremediation. Chemosphere 269, 128765. doi: 10.1016/j.chemosphere.2020.128765 33143888

[B40] Okkeri J. Haltia T. (1999). Expression and mutagenesis of ZntA, a zinc-transporting p-type ATPase from *Escherichia coli* . Biochemistry. 38 (42), 14109–14116. doi: 10.1021/bi9913956 10529259

[B41] Okkeri J. Haltia T. (2006). The metal-binding sites of the zinc-transporting p-type ATPase of *Escherichia coli.* Lys693 and Asp714 in the seventh and eighth transmembrane segments of ZntA contribute to the coupling of metal binding and ATPase activity. Biochim. Biophys. Acta 1757 (11), 1485–1495. doi: 10.1016/j.bbabio.2006.06.008 16890908

[B42] Olesen J. M. Bascompte J. Dupont Y. L. Jordano P. (2007). The modularity of pollination networks. PNAS. 104 (50), 19891–19896. doi: 10.1073/pnas.0706375104 18056808 PMC2148393

[B43] Petruk G. Roxo M. De Lise F. Mensitieri F. Notomista E. Wink M.. (2018). The marine gram-negative bacterium novosphingobium sp. PP1Y as a potential source of novel metabolites with antioxidant activity. Biotechnol. Lett. 41(2), 273–281. doi: 10.1007/s10529-018-02636-4 30542947

[B44] Qu H. Ma C. Xiao J. Li X. Wang S. Chen G. (2021). Co-Planting of *Quercus nuttallii*, *Quercus pagoda* with *Solanum nigrum* enhanced their phytoremediation potential to multi-metal contaminated soil. Int. J. Phytoremed. 23 (10), 1104–1112. doi: 10.1080/15226514.2021.1878105 33501836

[B45] Rizwan M. ElShamy M. M. Abdel-Aziz H. M. M. (2019). Assessment of trace element and macronutrient accumulation capacity of two native plant species in three different Egyptian mine areas for remediation of contaminated soils. Ecol. Indic. 106, 105463. doi: 10.1016/j.ecolind.2019.105463

[B46] Shrivastava M. Khandelwal A. Srivastava S. (2019). Heavy metal hyperaccumulator plants: the resource to understand the extreme adaptations of plants towards heavy metals. Plant-Metal Interact. 79–97. doi: 10.1007/978-3-030-20732-8_5

[B47] Subpiramaniyam S. (2021). *Portulaca oleracea* l. for phytoremediation and biomonitoring in metal-contaminated environments. Chemosphere 280, 130784. doi: 10.1016/j.chemosphere.2021.130784 33971418

[B48] Sungur A. Vural A. Gundogdu A. Soylak M. (2020). Effect of antimonite mineralization area on heavy metal contents and geochemical fractions of agricultural soils in gümüşhane province, Turkey. Catena 184, 104255. doi: 10.1016/j.catena.2019.104255

[B49] Sun H. Shao C. Jin Q. Li M. Zhang Z. Liang H.. (2022). Response of microbial community structure to chromium contamination in panax ginseng-growing soil. Environ. Sci. pollut. Res. 29, 61122–61134. doi: 10.1007/s11356-022-20187-0 35435557

[B50] Tang Z. Xi B. Huang C. Tan W. Xia X. Yang T.. (2019). Linking phytoavailability of heavy metals with microbial community dynamics during municipal sludge composting. Process Saf. Environ. Prot. 130, 288–296. doi: 10.1016/j.psep.2019.08.026

[B51] Tao J. Meng D. Qin C. Liu X. Liang Y. Xiao Y.. (2018). Integrated network analysis reveals the importance of microbial interactions for maize growth. Appl. Microbiol. Biotechnol. 102 (8), 3805–3818. doi: 10.1007/s00253-018-8837-4 29532103

[B52] Tian Z. Li G. Tang W. Zhu Q. Li X. Du C.. (2022). Role of *Sedum alfredii* and soil microbes in the remediation of ultra-high content heavy metals contaminated soil. gric Ecosyst. Environ. 339, 108090. doi: 10.1016/j.agee.2022.108090

[B53] Vaid N. Sudan J. Dave S. Mangla H. Pathak H. (2022). Insight into microbes and plants ability for bioremediation of heavy metals. Curr. Microbiol. 79 (5), 141. doi: 10.1007/s00284-022-02829-1 35320423

[B54] Walz J. Knoblauch C. Böhme L. Pfeiffer E.-M. (2017). Regulation of soil organic matter decomposition in permafrost-affected Siberian tundra soils - impact of oxygen availability, freezing and thawing, temperature, and labile organic matter. Soil Boil Biochem. 110, 34–43. doi: 10.1016/j.soilbio.2017.03.001

[B55] Wang X. Cui Y. Zhang X. Ju W. Duan C. Wang Y.. (2020). A novel extracellular enzyme stoichiometry method to evaluate soil heavy metal contamination: Evidence derived from microbial metabolic limitation. Sci. Total Environ. 738, 139709. doi: 10.1016/j.scitotenv.2020.139709 32590116

[B56] Wang K. Sitsel O. Meloni G. Autzen H. E. Andersson M. Klymchuk T.. (2014). Structure and mechanism of Zn^2+^-transporting p-type ATPases. Nature 514 (7523), 518–522. doi: 10.1038/nature13618 25132545 PMC4259247

[B57] Wood J. L. Tang C. Franks A. E. (2016). Microbial associated plant growth and heavy metal accumulation to improve phytoextraction of contaminated soils. Soil Boil Biochem. 103, 131–137. doi: 10.1016/j.soilbio.2016.08.021

[B58] Xiang M. Li Y. Yang J. Lei K. Li Y. Li F.. (2021). Heavy metal contamination risk assessment and correlation analysis of heavy metal contents in soil and crops. Environ. pollut. 278, 116911. doi: 10.1016/j.envpol.2021.116911 33740600

[B59] Xiao R. Shen F. Du J. Li R. Lahori A. H. Zhang Z. (2018). Screening of native plants from wasteland surrounding a zn smelter in feng county China, for phytoremediation. Ecotoxicol. Environ. Saf. 162, 178–183. doi: 10.1016/j.ecoenv.2018.06.095 29990729

[B60] Xuan Z. Baiquan Z. Hui L. Jing H. Lijuan J. Xian Z.. (2021). Soil heavy metals and phytoremediation by *Populus deltoides* alter the structure and function of bacterial community in mine ecosystems. Appl. Soil Ecol. L. 172, 104359. doi: 10.1016/j.apsoil.2021.104359

[B61] Xu Z. M. Zhang Y. X. Wang L. Liu C. G. Sun W. M. Wang Y. F.. (2022). Rhizobacteria communities reshaped by red mud based passivators is vital for reducing soil cd accumulation in edible amaranth. Sci. Total Environ. 826, 154002. doi: 10.1016/j.scitotenv.2022.154002 35231517

[B62] Yao Y. Zhang X. Huang Z. Li H. Huang J. Corti G.. (2022). A field study on the composition, structure, and function of endophytic bacterial community of *Robinia pseudoacacia* at a composite heavy metals tailing. Sci. Total Environ. 850, 157874. doi: 10.1016/j.scitotenv.2022.157874 35940266

[B63] Yin K. Wang Q. Lv M. Chen L. (2019). Microorganism remediation strategies towards heavy metals. Chem. Eng. J. 360, 1553–1563. doi: 10.1016/j.cej.2018.10.226

[B64] Yue Z. B. Li Q. Li C. C. Chen T. H. Wang J. (2015). Component analysis and heavy metal adsorption ability of extracellular polymeric substances (EPS) from sulfate reducing bacteria. Biores. Technol. 194, 399–402. doi: 10.1016/j.biortech.2015.07.042 26210529

[B65] Zerizghi T. Guo Q. Tian L. Wei R. Zhao C. (2022). An integrated approach to quantify ecological and human health risks of soil heavy metal contamination around coal mining area. Sci. Total Environ. 814, 152653. doi: 10.1016/j.scitotenv.2021.152653 34954188

[B66] Zhou S. Song Z. Li Z. Qiao R. Li M. Chen Y.. (2022). Mechanisms of nitrogen transformation driven by functional microbes during thermophilic fermentation in an ex situ fermentation system. Biores. Technol. 350, 126917. doi: 10.1016/j.biortech.2022.126917 35231599

