# Supplementary material for: Role of the rhizosphere bacterial community in assisting phytoremediation in a lead-zinc area
Source: Front Plant Sci. 2023 Jan 17;13:1106985. doi: 10.3389/fpls.2022.1106985 (PMC9982732; doi:10.3389/fpls.2022.1106985)
Supplement: Supplementary file 1 [file DataSheet_1.docx]

Supplementary Material

**Role of the rhizosphere bacterial community in assisting phytoremediation in a lead-zinc area**

Yunhua Xiao^1^, Liang Chen^1^, Chunxiao Li^1^, Jingjing Ma^1^, Rui Chen^1^, Bo Yang^1^, Gang Liu^1^, Shuming Liu ^2*^, Jun Fang^1*^

^1^College of Bioscience and Biotechnology, Hunan Agricultural University, Changsha, 410128, China

^2^ College of Chemical and Environmental Sciences, YiLi Normal University, YiLi, 835000, China

**^*^Corresponding Author:** Jun Fang ([fangjun1973@hunau.edu.cn](mailto:fangjun1973@hunau.edu.cn)) and Shuming Liu (liushuming@stu.hunau.edu.cn)

## 1.Supplementary Figures


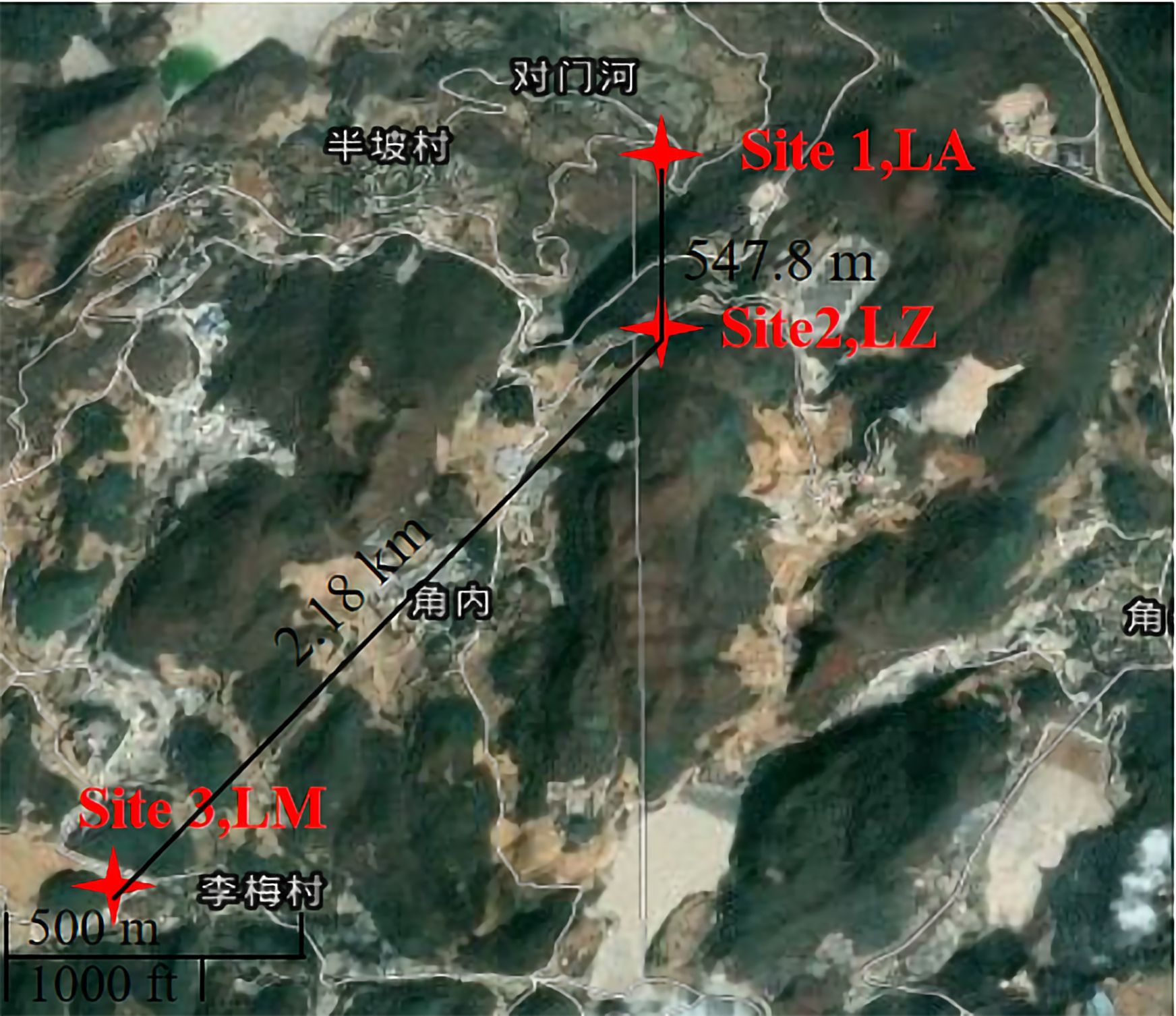


**Supplementary Figure 1.** Sampling coordinate point. LA, LZ, and LM represent the representative *Artemisia argyi*, *Boehmeria nivea*, and *Miscanthus floridulus* sampling sites, respectively.

##
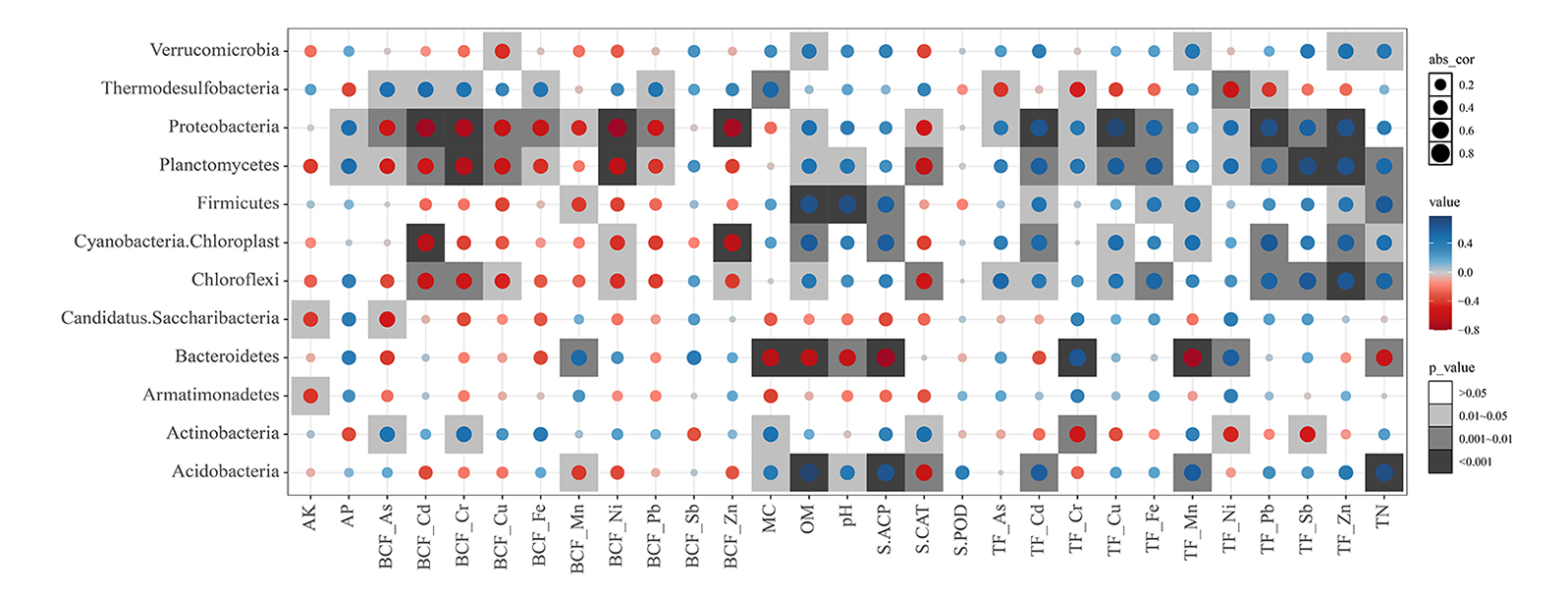


**Supplementary Figure** **2**. The correlation between environmental factors and the phyla.

## 2.Supplementary Table

## Supplementary Table 1. Dissimilarity test of three different plants

| Simple |  | LZ | LM |
| --- | --- | --- | --- |
| LA | ANOSIM | 0 | 0.714** |
|  | MRPP | 0.593*** | 0.630*** |
|  | Adonis | 0.445** | 0.348** |
| LZ | ANOSIM |  | 0.0891** |
|  | MRPP |  | 0.665*** |
|  | Adonis |  | 0.290** |

Note：The**/*** means significant difference (*p*＜0.05)

**Supplementary Table 2.** Network Indexes of three different plants

| **Network Indexes** | **LA(0.98)** | **LM(0.98)** | **LZ(0.98)** |
| --- | --- | --- | --- |
| Average clustering coefficient | 0.091 | 0.246 | 0.129 |
| Average degree | 3.425 | 3.977 | 5.062 |
| Harmonic geodesic distance | 3.309 | 3.301 | 2.916 |
| modularity | 0.581 | 0.568 | 0.489 |
| modules | 5 | 7 | 6 |
| R square of power-law | 0.586 | 0.591 | 0.633 |
| Total links | 137 | 175 | 329 |
| Total nodes | 80 | 88 | 130 |

Note：The (0.98) in the header represents the Cut off value

**Supplementary Table3.** Some of the functional genes related to heavy metals were significantly different

| functional genes | p-values | LA: mean rel. freq. (%) | LM: mean rel. freq. (%) | LZ: mean rel. freq. (%) |
| --- | --- | --- | --- | --- |
| copper chaperone NosL | 2.54E-09 | 0.127050371 | 0.039032697 | 0.033687046 |
| periplasmic iron binding protein | 9.45E-09 | 0.063368121 | 0.01437373 | 0.003487077 |
| zinc/manganese transport system ATP-binding protein | 3.31E-08 | 1.447794915 | 0.888137655 | 1.397348548 |
| complex iron-sulfur molybdoenzyme family reductase subunit alpha | 1.13E-07 | 0.071629125 | 0.008847712 | 0.002447458 |
| iron uptake system component EfeO | 1.75E-07 | 0.221414402 | 0.0981781 | 0.202091447 |
| iron complex transport system substrate-binding protein | 1.93E-07 | 2.323848438 | 1.629581682 | 1.563680815 |
| manganese transport system permease protein | 2.38E-07 | 0.298314891 | 0.198338493 | 0.102896376 |
| manganese-dependent inorganic pyrophosphatase [EC:3.6.1.1] | 2.43E-07 | 0.07218665 | 0.009998712 | 0.010418831 |
| zinc transport system ATP-binding protein [EC:3.6.3.-] | 4.92E-07 | 2.066834737 | 1.254840136 | 1.795137101 |
| zinc transport system permease protein | 5.45E-07 | 0.341212867 | 0.172114798 | 0.208115082 |
| small conductance mechanosensitive channel | 9.32E-07 | 0.36245209 | 0.19376043 | 0.126266207 |
| iron complex transport system ATP-binding protein [EC:3.6.3.34] | 1.78E-06 | 9.350254971 | 7.477652612 | 9.211698865 |
| zinc transport system substrate-binding protein | 1.93E-06 | 0.346874472 | 0.18975381 | 0.183487626 |
| iron complex transport system permease protein | 2.92E-06 | 2.310244872 | 1.560861339 | 1.544824294 |
| Rrf2 family transcriptional regulator, iron-responsive regulator | 3.11E-06 | 0.109284156 | 0.067839035 | 0.034579702 |
| manganese/zinc/iron transport system substrate-binding protein | 6.66E-06 | 0.03470655 | 0.011607868 | 0.003571848 |
| cobalt/nickel transport protein | 1.50E-05 | 0.160237589 | 0.052205927 | 0.13421565 |
| nitrogenase iron protein NifH [EC:1.18.6.1] | 1.58E-05 | 0.173842824 | 0.102084282 | 0.078452934 |
| large conductance mechanosensitive channel | 2.88E-05 | 0.699760216 | 0.622879742 | 0.562476675 |
| succinate dehydrogenase / fumarate reductase, iron-sulfur subunit [EC:1.3.5.1 1.3.5.4] | 7.60E-05 | 0.829976732 | 0.702688987 | 0.827662424 |
| two-component system, OmpR family, manganese sensing response regulator | 0.000103646 | 0.12644467 | 0.05725457 | 0.077654597 |
| manganese/iron transport system ATP-binding protein | 0.000268338 | 0.276252552 | 0.199753156 | 0.160273681 |
| high-affinity iron transporter | 0.000327527 | 0.362480075 | 0.266828475 | 0.250933409 |
| anaerobic carbon-monoxide dehydrogenase iron sulfur subunit | 0.000341631 | 0.067569892 | 0.022463941 | 0.027190135 |
| manganese/zinc-transporting P-type ATPase C [EC:3.6.3.-] | 0.00050103 | 0.054473037 | 0.019155898 | 0.042461116 |
| iron-hydrogenase subunit beta [EC:1.12.1.4] | 0.00099296 | 0.008003437 | 0.00050082 | 0.000184433 |
| 5-methyltetrahydrofolate corrinoid/iron sulfur protein methyltransferase [EC:2.1.1.258] | 0.003604115 | 0.00387435 | 7.38E-05 | 4.12E-05 |
| ferrous iron transport protein B | 0.004114234 | 0.477031051 | 0.358400898 | 0.313844535 |
| nickel transport protein | 7.17E-08 | 0.045334125 | 0.009625753 | 0.000963388 |
| zinc/manganese transport system substrate-binding protein | 1.86E-06 | 0.522714942 | 0.357020215 | 0.526026933 |
| nickel transport system substrate-binding protein | 1.95E-05 | 0.112481086 | 0.072318422 | 0.058688005 |
| nickel transport system permease protein | 7.67E-05 | 0.096848383 | 0.068449195 | 0.039148539 |
| manganese transport system ATP-binding protein | 9.85E-05 | 0.295418377 | 0.196329572 | 0.151947164 |
| putative iron-regulated protein | 0.000125928 | 0.099366964 | 0.087848969 | 0.042617574 |
| Fur family transcriptional regulator, iron response regulator | 0.00133531 | 0.312364511 | 0.212440773 | 0.278229749 |
| chloride channel protein, CIC family | 0.002677072 | 0.546428681 | 0.41970575 | 0.454134504 |
| CsoR family transcriptional regulator, copper-sensing transcriptional repressor | 0.002777435 | 0.713615798 | 0.456910129 | 0.587498279 |
| solute carrier family 30 (zinc transporter), member 9 | 0.010599106 | 0.009475204 | 0.002837273 | 0.00151469 |
| acid-activated urea channel | 0.014698142 | 0.011268559 | 0 | 0.002341087 |
| potassium-dependent mechanosensitive channel | 0.016962644 | 0.439753694 | 0.383603537 | 0.319892656 |
| anaerobic dimethyl sulfoxide reductase subunit B (DMSO reductase iron- sulfur subunit) | 0.019317607 | 0.075704533 | 0.030813544 | 0.07344349 |
| ArsR family transcriptional regulator, nickel/cobalt-responsive transcriptional repressor | 0.047934002 | 0.017272487 | 0.007118283 | 0.008819537 |
| zinc/manganese transport system permease protein | 6.34E-05 | 0.537982014 | 0.376328614 | 0.485946536 |
| iron/zinc/manganese/copper transport system ATP-binding protein | 7.86E-05 | 0.02462601 | 0.009037948 | 0.004854606 |
| glycolate oxidase iron-sulfur subunit | 0.000222191 | 0.28905636 | 0.220468728 | 0.290046929 |
| zinc D-Ala-D-Ala dipeptidase/carboxypeptidase [EC:3.4.13.22 3.4.17.14] | 0.035578918 | 0.001707314 | 5.27E-05 | 0 |
